# Comparison of Meniscal Cell-Mediated and Chondrocyte-Mediated Calcification

**DOI:** 10.2174/1874325001711010225

**Published:** 2017-03-31

**Authors:** Alex J. Kiraly, Andrea Roberts, Michael Cox, David Mauerhan, Edward Hanley, Yubo Sun

**Affiliations:** Department of Orthopaedic Surgery, Carolinas HealthCare System, Charlotte, NC, USA

**Keywords:** Articular cartilage, BCP, CPPD, Calcification crystals, Meniscus, OA

## Abstract

**Background:**

Chondrocytes have been traditionally thought to be responsible for calcium crystal deposits within osteoarthritic knees. Increasing recent experimental evidence suggests that menisci may also play a role. However, the calcifying potential of chondrocytes and meniscal cells derived from same OA patients, and the genes associated with meniscal calcification have never been fully examined.

**Objective:**

Examine and compare the calcifying potential of articular chondrocytes and meniscal cells derived from same OA patients and identify the calcium crystal type(s) and selected gene expression in OA menisci.

**Methods:**

Chondrocytes and meniscal cells were isolated from articular cartilage and menisci of OA patients undergoing total knee arthroplasty. Chondrocyte- and meniscal cell-mediated calcification was examined using both monolayer and micromass culture-based assays. Crustal types were examined with histological staining. Levels of Type X Collagen, MMP-13, and ANKH in OA menisci were examined using immunohistochemistry.

**Results:**

Primary human OA meniscal cells produced calcified deposits at a similar rate compared to OA chondrocytes in-vitro. Histological examinations indicate that both BCP crystals and CPPD crystals are present in the meniscal tissue. Type X collagen, MMP-13, and ANKH were found in human OA menisci and their levels increased with OA severity. In addition, type X collagen was co-localized with calcium crystals.

**Conclusion:**

These findings suggest that OA meniscal cells have a similar calcifying potential as OA chondrocytes, supporting a pathogenic role of OA menisci in OA.

## INTRODUCTION

Osteoarthritis (OA) is a degenerative joint disease that is characterized by cartilage degeneration, osteophyte formation, and synovial inflammation [1, 2]. At the current state, the associated changes involved in OA initiation and progression are not entirely understood. Many have thought previously that OA development was due to changes impacting articular cartilage alone; however, recent studies have implicated that OA is a disease of multifactorial components that involves the whole joint including the meniscus, synovium, and bone [3, 4]. The meniscus is a specialized tissue that aids in load transmission, shock absorption, and joint stability. There is increasing evidence suggesting that the knee meniscus may not be a passive bystander in OA, but may actually play a much larger part in conjunction with articular cartilage degradation [5, 6].

The formation of calcified articular crystals is a hallmark characteristic commonly associated with advanced OA [7]. These calcified deposits are found throughout the synovial fluid of approximately 65% of OA patients and in all of the cartilage samples obtained from OA patients undergoing knee replacement surgeries [8-10]. The two most commonly associated crystal types that have been identified throughout the joint structure of OA patients are basic calcium phosphate (BCP) and calcium pyrophosphate dehydrate (CPPD) crystals [11, 12]. BCP crystals include hydroxyapatite, octacalcium phosphate, and tricalcium phosphate and are more prominently found in degenerative joints and has been shown previously that the quantity of articular BCP crystals correlates with the severity of cartilage degeneration [13-15]. On the other hand, CPPD crystals have been recognized to induce inflammation though activation of NLRP-3 inflammasomes and also linked to a host of clinical ailments including osteoarthritis [16, 17].

Traditionally, chondrocytes have been indicated to be responsible for the formation of articular cartilage crystals and may influence OA development [18]. Recent reports suggest that meniscal degeneration and calcification are correlated with articular cartilage degeneration in OA, suggesting that the meniscus plays a role in the development of knee OA [9, 14]. BCP crystals stimulated the expression of inflammatory cytokines such as interleukin-1β (IL-1β) and cyclooxygenase-2 (COX-) as well as matrix degrading enzymes matrix metalloproteinase (MMP)-1 and MMP-13, both widely known as a major player in OA cartilage erosion [19]. BCP crystals have been shown to play a role in cartilage degradation by digesting collagen type II, whereas CPPD crystals have been shown to be associated with up-regulation of ankylosis protein homolog (ANKH) protein [20, 21].

Meniscal calcification is prevalent among individuals with CPPD arthroplasty. Studies found that 86% of patients with CPPD disease displayed calcified mineral deposits in the meniscus and that calcification increased with age [22, 23]. In a previous study conducted in our laboratory, we showed that OA meniscal cells produced more calcium deposits in culture than normal meniscal cells and displayed elevated expression of several genes involved in biomineralization [24].

In the present study, we sought to examine and compare the calcifying potential of OA meniscal cells and chondrocytes isolated from the same matching OA patient(s) as well as compare the crystal type and proportion of deposition in the meniscus of these OA patients. Furthermore, we sought to investigate the expression of genes implicated by previous work in biomineralization and inflammation in the meniscus [24]. Determination of the role meniscal cells in articular crystals formation in OA may not only advance our understanding of the pathogenesis of OA but may also provide information valuable for the identification of new targets for OA interventions.

## MATERIALS AND METHODS

### Meniscal and Articular Cartilage Specimens

Meniscus and hyaline cartilage from the tibial plateau was collected from the same OA patients undergoing total knee arthroplasty. Specimens were collected with the approval of the authors’ Institutional Review Board at Carolinas Healthcare System. Informed consent was waived due to tissues being surgical waste of routine arthroplasty and no patient private information was collected. The medial meniscus was processed to remove unwanted fat and synovial tissue. The meniscus was then sectioned in the middle to produce two portions, one was used for experimentations and one was reserved until use. The anterior portion of the bisected half was used for meniscal cell preparation and the posterior portion for histology.

### Cell Preparation

Meniscal cells and chondrocytes were isolated as described previously [24]. Briefly, a piece of menisci tissue (approximately 20 mm wide) was excised from the center part of the anterior portion of bisected meniscus and minced into small pieces. These small pieces were cultured in a 100 mm plate in Dulbecco’s Modified Eagle’s Medium, 10% fetal bovine serum and antibiotic/antimycotic mixture. Medium was changed every 2 to 3 days until cells reached 80% confluency, when the cells were then split and seeded prior to experimentation. Early passages of cells (passage 2 or 3) were used since later passages have been shown to exhibit a fibroblast-like morphology and become less responsive to external stimuli. Chondrocytes were prepared from Hyaline articular cartilage derived from the same patient similarly.

### Adenosine-5’-triphosphate (ATP)-induced Calcium Deposit Assay

A ^45^calcium assay was used to detect calcification in chondrocyte- and meniscal cell-mediated calcification. Briefly, meniscal cells or chondrocytes from the same OA patients (passage 2 or 3) were cultured in a monolayer environment in a 24-well plate to 95 to 100% confluence. On the second day, media was replaced with serum-free media for 24 hours. The culture media was then replaced with media trace-labeled with 1 µCi/ml ^45^Ca. Immediately afterwards, ATP was added at a final concentration of 1 mM per well (triplicate). Cells cultured in medium without ATP were treated with β-glycerophosphate as a control. After 48 hours, culture media was removed and the cells were washed with Hank’s balanced salt solution followed by the addition of 0.1 N NaOH. Radioactivity of the lysate, count per minute (CPM), in each well was quantified using liquid scintigraphy. This experiment was repeated 3 times. This assay was repeated three times using chondrocytes and meniscal cells isolated from the same OA patient (n=3).

### Monolayer Meniscal Cell- and Chondrocyte-mediated Calcification in Chondrogenesis Medium

Alizarin red staining was used to detect calcification in chondrocytes and meniscal cells cultured in chondrogenesis and osteogenesis media. ATP has been shown to be hydrolyzed by the cells and provide the phosphate and pyrophosphate needed for crystal formation. Isolated meniscal cells or chondrocytes (passage 2 or 3) were cultured in a monolayer environment in a 48-well plate to 85-90% confluency. The next day, media was replaced with serum-free media for 24 hours. Afterwards, culture media was then replaced with StemPro^®^ Chondrogenesis or Osteogenesis differentiation media with or without ATP (1 mM). These cells were cultured for 14 days with fresh differentiation media being replaced every three days. After 14 days, the media was removed. Calcium mineral deposition was examined by staining with 2% alizarin red at pH 4.1-4.3. Alizarin red stains were extracted from each well with 200 μl acidic water (0.1 mM hydrogen chloride) and quantified by reading at 405 nm using a microplate reader. This experiment was repeated 3 times. Pictures were taken using Sony Imaging software with an inverted microscope.

### Calcification in Micromass Culture

OA meniscal cells or chondrocytes were harvested from 100 mm culture plates and suspended in culture medium containing 10% serum. For preparing a micromass, a droplet of the cell suspension containing 6 x 10^6^ chondrocytes was placed in a well of a 24-well plate. After placing all droplets, the plate was incubated for 4 hours at 37°C in a tissue culture incubator and then fed with chondrogenic or osteogenic media (StemPro^®^ Chondrogenesis and Osteogenesis kits, Thermo Fisher Scientific) with or without ATP (1 Mm). The plate was incubated for 48 hours, followed by changing the media every 2 to 3 days for a total of 14 days. At the end of the experimental period, each well containing a micromass was rinsed twice with 500 µL of Hank’s balanced salt solution. Two drops of eosin were added to each well. Five minutes later, eosin was aspirated off and micromass clusters were transferred to a strip of filter paper on top of an ethanol-soaked sponge within a plastic histology specimen cassette. The cassettes sat in 10% formalin solution for one hour. These micromasses underwent routine paraffin embedding. Sections were cut at 5 µm thick and stained with alizarin red.

### Calcium Mineral Deposition in OA Menisci

The posterior medial meniscus of 10 OA patients was collected and fixed in 10% neutral buffered formalin overnight and then transferred to 70% ethanol until experimentation. The posterior portion of the medial menisci was stained with either alizarin red for total mineral or stained with eosin y to identify CPPD crystal types [25]. The percent of BCP and CPPD crystals per area was measured using OsteoMeasureXP software_._

### Immunohistochemistry

Paraffin sections of the posterior portion of the medial meniscus were sectioned at 4μm, collected on PLUS slides (Cardinal Health, Dublin, OH) and dried at 60^o^C. Sections were deparaffinized in xylene (Cardinal) and rehydrated through graded alcohols (AAPER, Shelbyville, KY) to distilled water. Endogenous peroxidase was blocked using 3% H_2_O_2_ (Sigma, St Louis, MO). Sections were incubated for one hour with MMP-13, ANKH, and Collagen X, (Santa Cruz Biotechnologies, Santa Cruz, CA) at a dilution of 1:100. Slides were then treated with Vector ImmPress anti-mouse IgG reagent (Vector Laboratories, Burlingame, CA) for 30 minutes and DAB (Dako, Carpinteria, CA) for 5 minutes. Slides were rinsed in water, counterstained with light green, dehydrated, cleared, and mounted with resinous mounting media. Human liver was used as a positive control and Mouse IgG (Dako) was used as a negative control.

### Statistics

Statistical analysis was analyzed using Student’s t-test. All results were presented as mean ± standard deviation (SD). For each analysis, p < 0.05 was considered statistically significant. Statistical analysis was performed using GraphPad Prism Software (La Jolla, CA).

## RESULTS

### OA Meniscal Cell- and Chondrocytes-mediated Calcification

**Fig. (1):**
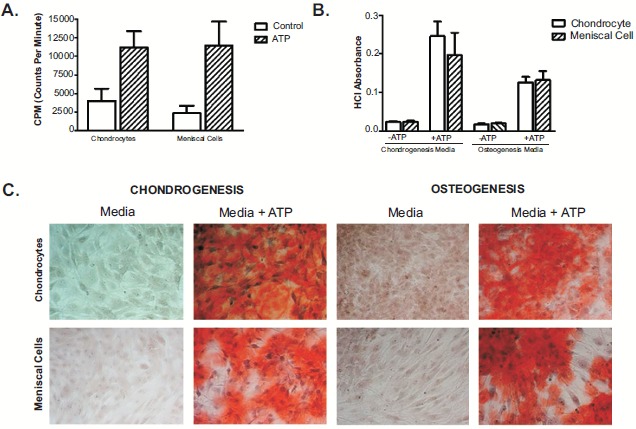
ATP-induced calcium deposition in monolayer culture of osteoarthritis chondrocytes and meniscal cells derived from the same patients. Fig. (**1A**), meniscal cells and chondrocytes induced calcification measured by uptake of ^45^Ca (n=3). Fig. (**1B**), cells were cultured in chondrogenesis and osteogenesis media and treated with extracellular ATP (n=5). Alizarin red staining was quantified (1B) to measure the absorbance correlated to calcium deposition after visualized (1C) using light microscopy.

^45^Calcium Assay indicated that OA meniscal cells induced ^45^calcium deposition at a similar rate as the OA articular chondrocytes derived from the same patient (Fig. **1A**). When cultured in chondrogenic media, OA chondrocytes appeared to produce a little more calcium mineral than the OA meniscal cells derived from the OA patient; however, when cultured in osteogenic media, OA meniscal cells appeared to produce a little slightly more calcium mineral than the OA chondrocytes (Fig. **1B**). In Fig. (**1C**), images show alizarin red staining of OA meniscal cells and chondrocytes in monolayer culture fed with chondrogenesis or osteogenesis media. These findings indicated that OA meniscal cells displayed a similar calcifying potential as the OA chondrocytes.

### Calcification in Micromass Culture

Next, we seeded primary OA chondrocytes and meniscal cells in a micromass culture to mimic a 3D environment. Calcification in this condition with and without the presence of ATP was examined. As shown in Fig. (**2**), both OA meniscal cells and chondrocytes under 3D environment cultured in chondrogenesis media produced easily detectable mineral deposits without ATP treatment, indicating that OA meniscal cell and chondrocytes in vivo-like condition, are prone to pathological calcification. In the presence of ATP, both cell types showed a substantial increase in alizarin red staining, signifying an increase in calcified deposits throughout the micromass culture. Again OA meniscal cells and OA chondrocytes displayed a similar calcifying potential.

### Calcium Mineral Deposition in OA Menisci

**Fig. (3):**
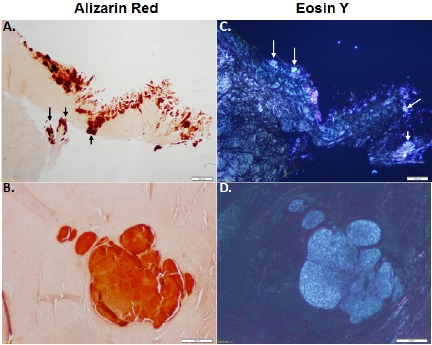
Meniscus tissue from OA patients (n=15) stained with alizarin red (A, B) and eosin Y (C, D). Alizarin red stains for all calcium crystals in comparison to eosin Y, which only stains for CPPD crystals under polarizing light microscopy. White arrows represent eosin Y positive CPPD crystals, while black arrows represent calcium crystals that are positive for alizarin red and not eosin Y (BCP). Scale bar = 500 µm (top), 200 µm (bottom).

In order to distinguish between crystal type in the meniscus, the medial menisci isolated from OA patients were stained with alizarin red and eosin Y. CPPD crystals can be exclusively detected using eosin y staining and visualized using polarizing lenses. Alizarin red does not exclusively stain BCP crystals and stains all calcium crystals. As shown in Figs. (**3A**, **B**), alizarin red staining shows the presence of mineral deposition in OA menisci. Eosin Y staining, visualized under polarizing light microscopy, shows that CPPD crystals were co-localized with the minerals stained with alizarin red (Figs. **3C**, **D**). Examinations also revealed that some areas were positive for alizarin red but negative for eosin Y staining. It appeared that approximately 80% of the areas stained positive with alizarin red was positive with eosin Y, indicating widespread deposition of CPPD crystal in OA menisci.

### Immunohistochemistry

**Fig. (4):**
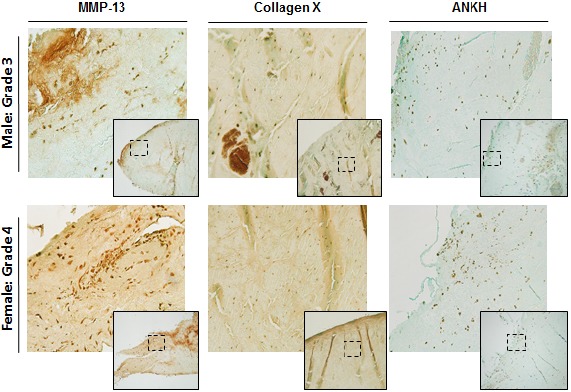
Representative images of MMP-13, Collagen X, and ANKH expression from the meniscus of nine OA patients undergoing total knee arthroplasty. Images on the top are from a Male patient with a Grade 3 meniscus whereas the bottom images are from a Female patient with a Grade 4 meniscus. Grading was conducted by the surgeon at the time of extraction. Images in the smaller box were taken with 4x objective and larger images are of the dashed outline box taken with the 10x objective.

We examined and compared the levels of MMP-13, Collagen X, and ANKH proteins in the menisci of OA patients (Fig. **4**). As shown, OA menisci displayed high levels of MMP-13 and Collagen X throughout the entire menisci. ANKH was also positive grade 4 meniscus.

We examined consecutive sections alternatively with type X collagen antibody and alizarin red to determine whether type X collagen and crystals were co-localized. As shown in Fig. (**5**), immunostaining of type X collagen were indeed co-localized with alizarin red staining of calcium mineral within the OA menisci, indicating that meniscal cell hypertrophy played a key role in meniscal crystal deposition.

## DISCUSSION

The presence of articular crystals in knee joint fluid is a hallmark of end-stage of OA. Degenerated cartilage (shedding crystals into synovial fluid) or chondrocytes have been traditionally indicated to be the source of articular crystals. Here we tested the hypothesis that degenerated menisci or meniscal cells may contribute to the formation of articular calcium crystals. We have previously showed that OA meniscal cells were capable to produce calcium minerals [24], however, the meniscal cells and chondrocytes examined in the previous study were not from the same OA patients. The relative calcifying potential of meniscal cells and chondrocytes derived from same OA patients was unknown. In this study we examined meniscal cells and chondrocytes derived from same OA patients using multiple assays. It is known that OA synovial fluid contains high level of ATP and factors promoting the formation of osteophytes. Therefore, we examined these cells using both monolayer and micromass cultures at the presence of ATP and factors that promote osteogenesis or chondrogenesis to mimic in vivo-like conditions. We demonstrated that meniscal cells and chondrocytes derived from OA patients produced calcium crystals at the relatively same rate. Meniscal calcification was stimulated with ATP in chondrogenic or osteogenic media that contains factors promoting chondrogenesis or osteogenesis and prevent these cells differentiation into fibroblast-like cells. These findings indicate that meniscal cells, like chondrocytes, may also play a role in the formation of articular crystals in vivo condition within OA knee joints.

Identification of the specific crystal type produced in the meniscus was not established in our previous study. In this study, we performed both alizarin red staining and eosin Y staining to estimate the relative amounts of BCP crystals and CPPD crystals within the OA menisci. CPPD crystals can be exclusively detected using eosin Y staining and polarizing lenses [25]. Alizarin red stains all calcium crystals types. In the 10 menisci we examined, approximately 80% of the area stained with alizarin red contained CPPD crystals, as indicated by the eosin Y staining. This finding demonstrates that both crystal types are present within OA menisci and that a large amount of crystals within the OA menisci appeared to be CPPD crystals.

In addition to calcium deposits, we examined the levels of MMP-13, type X collagen, and ANKH in the menisci derived from several patients. These three proteins have been implicated in OA development, crystal formation, and hypertrophic differentiation of OA chondrocytes. As expected, we saw high level of MMP-13 throughout the meniscus. Several previous studies have demonstrated that MMP-13 plays an important role in articular cartilage degeneration [26-28]. Our finding indicates that MMP-13 also plays a role in meniscal degeneration and potentially in meniscal calcification as well because MMP-13 is a marker of chondrocyte hypertrophy. Type X collagen, another marker of chondrocyte hypertrophy, was also highly expressed in OA meniscus. This suggests that meniscal cells underwent hypertrophic differentiation with disease progression similar to OA chondrocyte in hyaline cartilage [9]. Furthermore, we have previously shown that the expression of ANKH is upregulated in OA meniscal cells in comparison to normal non-OA meniscal cells [29]. Immunostaining revealed high level of ANKH within the OA menisci, indicating that ANKH may play a role in the formation of CPPD crystal in OA menisci.

## CONCLUSION

These findings, combined with our previous results that calcium deposits were present in all OA menisci and that OA meniscal cells induced much more calcium deposition than normal meniscal cells, will have a significant impact on our understanding of initiation and promotion of OA. Our overall findings suggest that OA is a disease due to articular cartilage degeneration, articular calcification, and meniscal calcification. Calcium deposits found throughout the meniscus can alter the biomechanical properties of the meniscus resulting in upregulation of inflammatory genes and cytokines as well as matrix metalloproteases that contribute to the initiation and progression of OA. Additionally, this new insight reveals a new potential therapeutic target for directly treating OA and not just the symptoms associated with OA.

### OA-Osteoarthritis

In summary, OA meniscal cells may also play an important role in the formation of articular crystals within OA knee joints. Similar to OA chondrocytes, OA meniscal cells undergo hypertrophic differentiation and produce not only calcium crystals but also a large amount of matrix degrading enzyme of MMP-13. These findings provide further support that OA is not just a cartilage disease but a whole joint disease.

## Figures and Tables

**Fig. (2):**
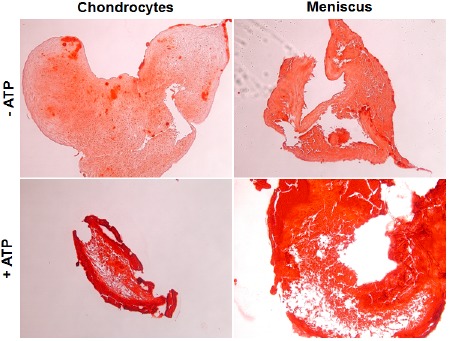
Micromass culture of both chondrocytes and meniscal cells isolated from OA patients. Alizarin red staining shows an increase in calcium deposition in both chondrocytes and meniscal cells of the same patient when stimulated with ATP.

**Fig. (5):**
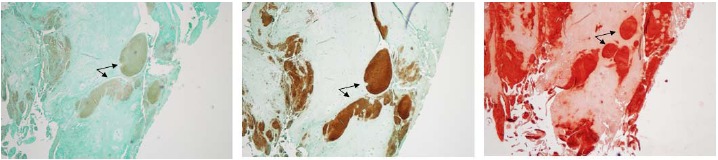
Histological examination of calcified deposits from Male Grade 3 Meniscus extracted after surgery. Serial sections were cut and stained. Left image, negative control. Middle image, stained with type X collagen. Right image, alizarin red stain. Arrows indicate an example of calcified deposits due to cellular hypertrophy. Images were taken with 4x objective.

## LIST OF ABBREVIATIONS

ANKH: = Ankylosis progressive analog
ATP: = Adenosine Triphosphate
BCP: = Basic Calcium Phosphate
COX-2: = Cyclooxygenase 2
CPM: = Counts per minute
CPPD: = Calcium Pyrophosphate Dehydrate
DAB - 3: = 3’-diaminobenzidine
MMP: = Matrix Metalloproteinase
OA: = Osteoarthritis
